# Fructose metabolism in *Chromohalobacter salexigens*: interplay between the Embden–Meyerhof–Parnas and Entner–Doudoroff pathways

**DOI:** 10.1186/s12934-019-1178-x

**Published:** 2019-08-13

**Authors:** José M. Pastor, Nuno Borges, Juan P. Pagán, Sara Castaño-Cerezo, Laszlo N. Csonka, Bradley W. Goodner, Kathryn A. Reynolds, Luís G. Gonçalves, Montserrat Argandoña, Joaquín J. Nieto, Carmen Vargas, Vicente Bernal, Manuel Cánovas

**Affiliations:** 1 Dept. de Bioquímica y Biología Molecular B e Inmunología. Facultad de Química. Campus Regional de Excelencia Internacional “Campus Mare Nostrum”, Universidad de Murcia, 30100 Murcia, Spain; 2 Instituto de Tecnologia Química e Biologica, Universidade Nova de Lisboa, Oeiras, Portugal; 3 Dept. of Biological Sciences, Purdue University, West Lafayette, IN 47907-2064 USA; 4 Dept. of Biology, Hiram College, Hiram, OH 44234 USA; 5 Dept. of Microbiology and Parasitology, University of Seville, 41012 Seville, Spain; 6 Centro de Tecnología de Repsol, Repsol S.A. Calle Agustín de Betancourt, s/n, 28935 Móstoles, Spain

**Keywords:** *Chromohalobacter salexigens*, Fructose metabolism, Pyrophosphate-dependent 6-phosphofructokinase, Fructose bisphosphatase, Ectoines, Entner–Doudoroff pathway, ^13^C-NMR

## Abstract

**Background:**

The halophilic bacterium *Chromohalobacter salexigens* metabolizes glucose exclusively through the Entner–Doudoroff (ED) pathway, an adaptation which results in inefficient growth, with significant carbon overflow, especially at low salinity. Preliminary analysis of *C. salexigens* genome suggests that fructose metabolism could proceed through the Entner–Doudoroff and Embden–Meyerhof–Parnas (EMP) pathways. In order to thrive at high salinity, this bacterium relies on the biosynthesis and accumulation of ectoines as major compatible solutes. This metabolic pathway imposes a high metabolic burden due to the consumption of a relevant proportion of cellular resources, including both energy molecules (NADPH and ATP) and carbon building blocks. Therefore, the existence of more than one glycolytic pathway with different stoichiometries may be an advantage for *C. salexigens.* The aim of this work is to experimentally characterize the metabolism of fructose in *C. salexigens*.

**Results:**

Fructose metabolism was analyzed using in silico genome analysis, RT-PCR, isotopic labeling, and genetic approaches. During growth on fructose as the sole carbon source, carbon overflow was not observed in a wide range of salt concentrations, and higher biomass yields were reached. We unveiled the initial steps of the two pathways for fructose incorporation and their links to central metabolism. While glucose is metabolized exclusively through the Entner–Doudoroff (ED) pathway, fructose is also partially metabolized by the Embden–Meyerhof–Parnas (EMP) route. Tracking isotopic label from [1-^13^C] fructose to ectoines revealed that 81% and 19% of the fructose were metabolized through ED and EMP-like routes, respectively. Activities of enzymes from both routes were demonstrated in vitro by ^31^P-NMR. Genes encoding predicted fructokinase and 1-phosphofructokinase were cloned and the activities of their protein products were confirmed. Importantly, the protein encoded by *csal1534* gene functions as fructose bisphosphatase, although it had been annotated previously as pyrophosphate-dependent phosphofructokinase. The gluconeogenic rather than glycolytic role of this enzyme in vivo is in agreement with the lack of 6-phosphofructokinase activity previously described.

**Conclusions:**

Overall, this study shows that *C. salexigens* possesses a greater metabolic flexibility for fructose catabolism, the ED and EMP pathways contributing to a fine balancing of energy and biosynthetic demands and, subsequently, to a more efficient metabolism.

**Electronic supplementary material:**

The online version of this article (10.1186/s12934-019-1178-x) contains supplementary material, which is available to authorized users.

## Introduction

*Chromohalobacter salexigens* is an aerobic halophilic bacterium that accumulates the compatible solutes ectoine and hydroxyectoine (ectoines) in response to salt and heat stress, respectively [1, 2]. These compounds have gained much biotechnological interest due to the increasing number of applications in the bio-stabilization of cells and macromolecules, including skin care and treatment of neurodegenerative diseases [3].

In nature, ectoines, and other compatible solutes must be either taken up from the environment or synthesized de novo [4–6]. Specifically, the biosynthesis of ectoines imposes a metabolic burden due to the consumption of a relevant proportion of cellular resources, including both energy molecules (NADPH and ATP) and carbon building blocks [7] that must be met by the halophilic microorganism [8]. For this reason, halophiles have evolved specific metabolic adaptations that are of high interest, both for basic and applied science [3, 9, 10]. In a recent paper, we described the metabolism of glucose in *C. salexigens*. This bacterium has evolved specific metabolic adaptations that increase its chances to survive in highly demanding environments. For instance, high anaplerotic fluxes are needed to sustain the synthesis of ectoines at the expense of the tricarboxylic acid (TCA) cycle intermediate oxaloacetate. *C. salexigens* solves this issue with a high pyruvate carboxylase activity, thus replenishing the oxaloacetate pool at the expense of pyruvate [11]. In addition, the extensive degree of metabolic adaptation to growth at high salinity has derived in a significant rigidity of the pyruvate-acetyl CoA-oxaloacetate metabolic node, which, in turn, leads to inefficient metabolism of glucose, and high pyruvate and acetate overflow at low salinity [11, 12].

Glycolysis is the route for initial glucose catabolism in many microorganisms [13]. In bacteria, glycolysis occurs as several pathways, which share some enzymatic steps and differ in the overall redox and energy balances. Depending on the microorganism, carbon source or environmental conditions, these pathways may be concurrent. The Embden–Meyerhof–Parnas (EMP) pathway consists of ten enzymatic steps and yields two moles of pyruvate, NADH and ATP, each, per mol of glucose (Additional file 1: Figure S1). The Entner–Doudoroff (ED) pathway is an alternative route for glucose catabolism which is present in many microbes [14]. It comprises two specific steps which are catalyzed by phosphogluconate dehydratase (Edd) and 2-keto-3-deoxygluconate-6-phosphate aldolase (Eda) enzymes; and the rest of steps are shared with the oxidative pentose phosphate (PP) and the EMP pathway. Overall, glucose oxidation through the ED pathway variant yields 2 moles of pyruvate, but only 1 mole of NADH, NADPH and ATP per mol of glucose (Additional file 1: Figure S1). The major differences between both pathways are: (i) the mechanism of glucose phosphorylation, (ii) the set of enzymes used and (iii) the overall energy yield [15, 16]. In fact, the ED route is thermodynamically more favorable than the EMP route, despite being less efficient in terms of energy harvest. Additionally, the ED route is more efficient than the EMP pathway in terms of enzymatic protein mass needed to metabolize a given amount of glucose [17, 18].

In *E. coli* and many bacteria, both the ED and EMP pathways coexist, and their respective contribution to overall glucose oxidation can be discerned using labeled substrates [19]. However, many bacteria possess only the ED pathway, frequently due to the lack of 6-phosphofructokinase, one of the regulatory enzymes of the EMP pathway. This is the case of *Pseudomonas* species and *C. salexigens* [11, 20]. The preference of *C. salexigens* for the ED pathway might be related to the production of NADPH, which is needed for the synthesis of ectoines [11]. Thus, the ED pathway may play a key role in successful adaptation to high salinity conditions [21, 22].

A preliminary analysis of the genome of *C. salexigens* revealed the presence of genes encoding a fructose-specific phosphoenolpyruvate phosphotransferase transporter system (PTS^Fru^) [23]. PTS^Fru^ encodes for a fructose specific transporter [24]. This finding suggested that *C. salexigens* might be able to assimilate fructose through an EMP-like pathway, since the transport of sugars through PTS is not compatible with their exclusive metabolism via the ED pathway [25]. This incompatibility is explained by the fact that the ED route yields only one mol of PEP per mol of hexose, unlike the EMP route, which yields 2 moles of this metabolite. Therefore, fructose transport through PTS^Fru^, which consumes 1 mol of phosphoenolpyruvate (PEP) per mol of hexose transported, would quantitatively seize all the PEP generated via the ED route. This would be incompatible with efficient growth, since PEP is essential as building block for the synthesis of anabolic precursors (e.g. aromatic amino acids, *N*-acetylmuramic acid). Therefore, the existence of PTS^Fru^ makes the metabolism of fructose as sole carbon source by *C. salexigens* through the combination of the EMP and ED pathways not only advantageous, but compulsory.

In this work, we present a detailed molecular and physiological characterization of the metabolism of fructose in *C. salexigens.* This includes the in silico reconstruction and transcriptional analysis of the pathways involved, use of genomic approaches to understand the roles of genes, in vitro assessment of fructose metabolic enzymes using ^31^P-NMR and spectrophotometric assays, as well as in vivo determination of the contribution of the EMP and ED pathways to fructose catabolism. The preferential use of the ED pathway for fructose metabolism is discussed in the context of energy demands and adaptation to adverse environmental conditions.

## Results

### Growth of *C. salexigens* in fructose minimal medium. Biomass formation and metabolic overflow

**Fig. 1:**
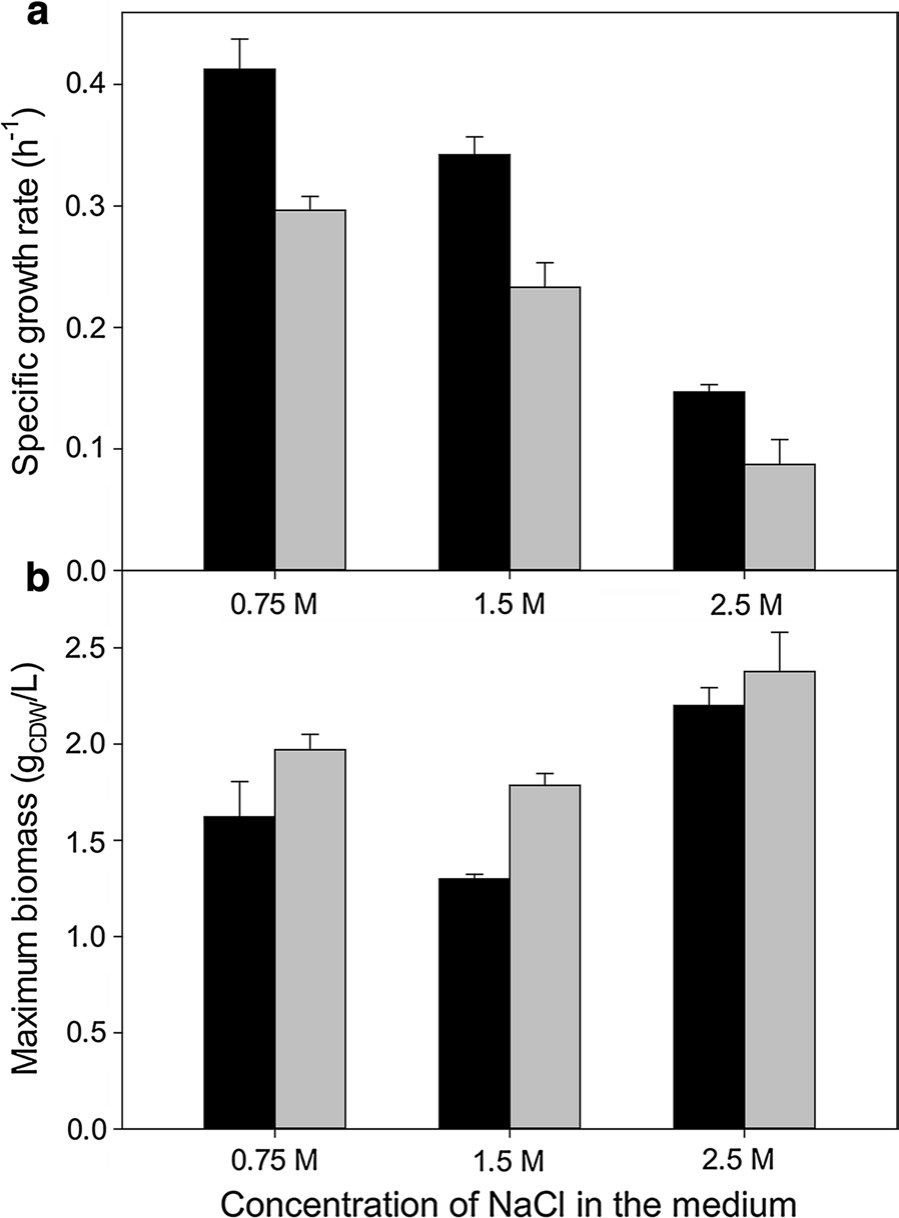
Specific growth rates (**a**) and maximal biomass yields (**b**) from batch cultures grown in glucose and fructose. Cultures were grown at 37 °C in M63 minimal medium with 20 mM glucose (dark bars) or 20 mM fructose (light bars) as the sole carbon source in 0.75, 1.5 or 2.5 M NaCl. All measurements are the average of at least three independent cultures. See “Methods” section for details

Growth of *C. salexigens* with fructose or glucose as sole carbon sources was compared as a function of salinity. As shown previously [11], maximum biomass yield increased at high salinities with glucose as the carbon source, while growth rate decreased by 60% from 0.75 M NaCl (optimal salinity for maximum growth rate) to 2.5 M NaCl (optimal salinity for biomass synthesis). The growth rate in fructose medium was 30–40% lower than that in glucose at any salinity tested (Fig. 1a), while the maximum biomass yield was higher than that in glucose cultures, except at 2.5 M NaCl, where both were similar (Fig. 1b). Production of ectoines was assessed in parallel cultures grown in 2.5 M NaCl; intracellular ectoines accumulation was higher in the fructose-grown cultures (1.419 ± 0.087 mmol/gCDW) as compared to glucose grown cultures (1.195 ± 0.037 mmol/gCDW).

Pyruvate and acetate were not detected in fructose culture supernatants. The absence of carbon overflow in fructose cultures was in agreement with the higher biomass yield. In addition, no significant difference in the biomass yield from either fructose or glucose was observed at 2.5 M NaCl. Under these conditions, carbon overflow in glucose cultures was very low, as previously described [11], thus approaching the behavior observed in fructose cultures at all salinities assayed.

**Fig. 2:**
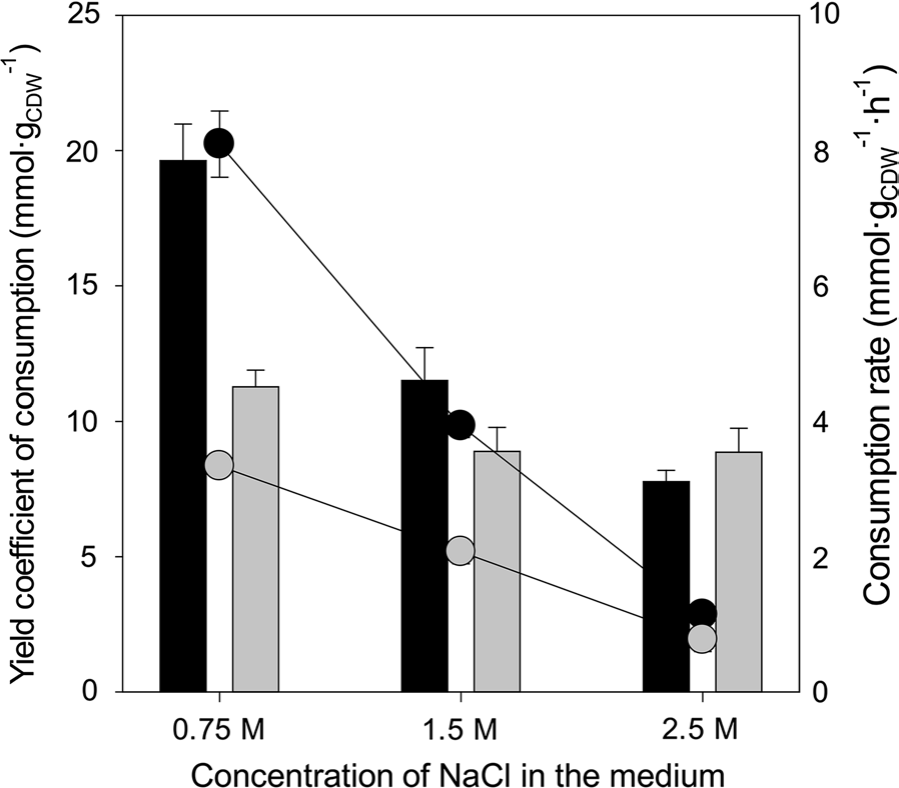
Glucose and fructose consumption efficiency. The yield coefficients of glucose (1/Y_X/Glc_, dark bars) and fructose consumption (Y_X/Fru_, light bars) at different salt concentrations are shown. The consumption rate for both carbon sources is denoted by circles following the same color code. Cultures were grown at 37 °C in M63 minimal medium with 20 mM glucose or fructose as the sole carbon source in 0.75, 1.5 or 2.5 M NaCl. All measurements are the average of at least three independent cultures

To understand central metabolism of *C. salexigens* growing on fructose as the sole carbon source, we evaluated the yield coefficient of carbon source consumption for biomass production. 1/Y_X/S_ represents the amount of carbon source consumed per unit of biomass synthesized by the culture, which decreased at high salinity, therefore suggesting that the efficiency of carbon source consumption increased with salinity for both carbon sources. This observed trend was much stronger in the case of glucose. Accordingly, the rate of carbon source consumption was lower in the case of fructose in all conditions assayed (Fig. 2).

Overall, growth at low salt concentration was more efficient in fructose, while growth parameters at high salinity were similar. These phenotypic differences correlated well with the presence of overflow metabolism.

### Reconstruction of fructose metabolic pathways in *C. salexigens* from genomic, transcriptomic and enzyme activity analysis

**Fig. 3:**
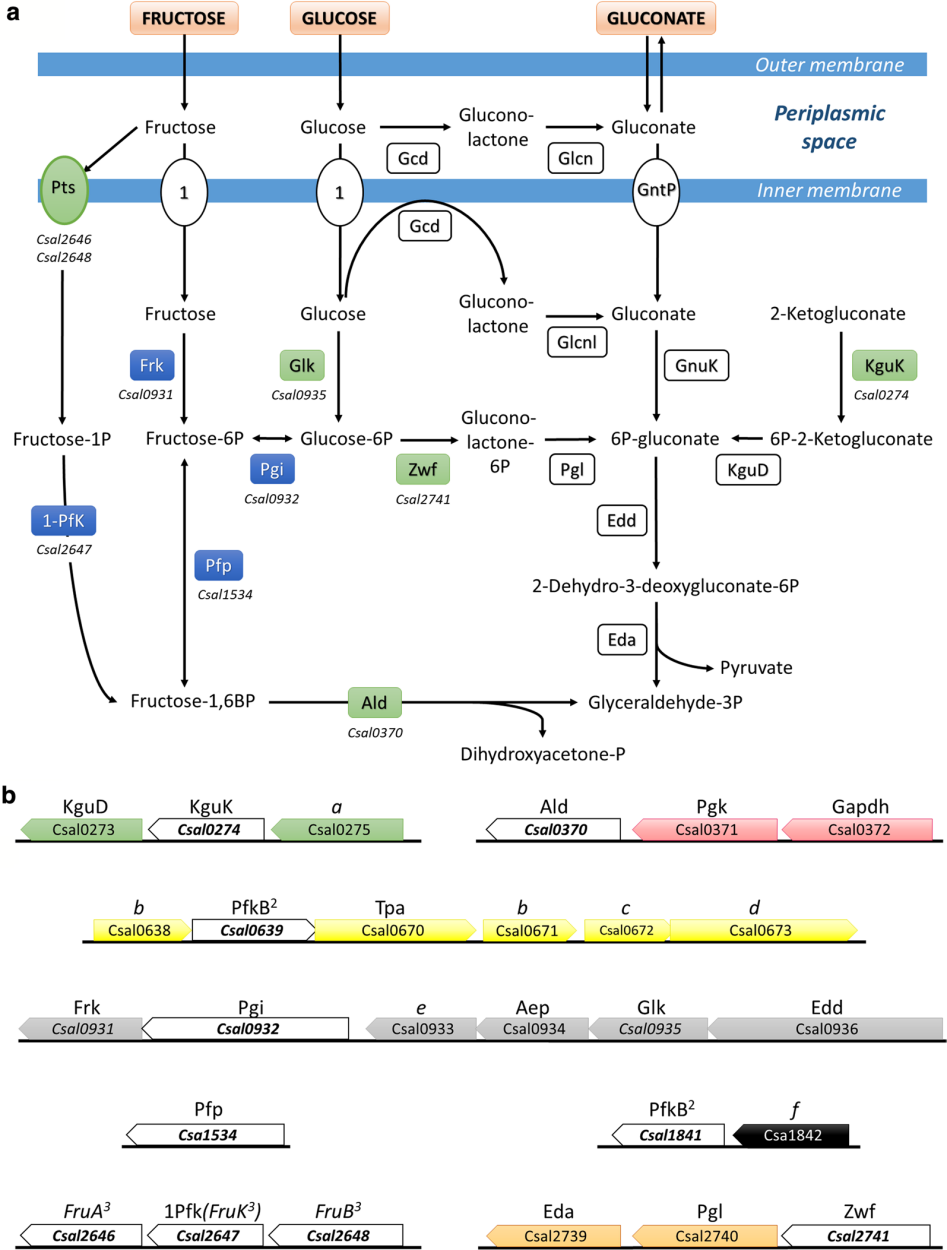
**a** Pathways for fructose, glucose and gluconate assimilation in *C. salexigens*. The proposed pathways are based on previous in silico and phylogenetic studies comparing *C. salexigens* with *P. putida* and other closely related species. The two proposed fructose assimilation pathways, are supported by ^13^C-, and ^31^P-NMR results and RT-PCR and heterologous expression studies from the present work. Names of enzymes and transporters are shown in rectangles and ellipses, respectively. Enzyme activities confirmed by ^31^P-NMR and included in RT-PCR expression studies are shown in blue boxes; ORFs from RT-PCR studies not assayed/not detected by ^31^P-NMR assays are shown in green boxes. The rest of proposed enzymes and transporters are shown in colourless boxes. **b** Gene clusters and operons related to fructose and glucose assimilation pathways in *C. salexigens*. Above each ORF, the name of the most plausible functional assignment is shown. ORFs included in RT-PCR studies are in colourless rectangles. 1Pfk: 1-phosphofructokinase; Aep: aldose-1-epimerase; Ald: fructose-1,6-bisphosphate aldolase; Eda: 2-keto-3-deoxy-6-phosphogluconate aldolase; Edd: 6-phosphogluconate dehydratase; Frk: fructokinase; Gapdh: glyceraldehyde-3-phosphate dehydrogenase; Gcd: glucose dehydrogenase; Glcnl: gluconolactonase; Glk: glucokinase; GntP: gluconate transporter; KguD: 6-phosphogluconate dehydrogenase, KguK: 2-ketogluconate kinase; 2-keto-6-phosphogluconate reductase; KguT: 2-ketogluconate transporter; Pgi: phosphoglucose isomerase; Pgl: 6-phosphogluconolactonase; Pgk: phosphoglycerate kinase; Pts: phosphotransferase transport system (fructose specific); Tpa: tagatose-6-phosphate aldolase; Zwf, glucose-6-phosphate dehydrogenase. ^1^Sugar facilitators or ABC transporters (genes not assigned yet). ^2^The two remaining ORFs that are still assigned to PfkB on the basis of similarity of their predicted sequences to authentic phosphofructokinases, but without any further experimental evidence. ^3^*fruBKA* operon. The following ORFs bear a wide assignment based on automated annotation: *a*, LacI family transcriptional regulator; *b*, short chain dehydrogenase/reductase; *c*, putative fructose transport system kinase; *d*, mannitol dehydrogenase-like protein; *e*, aldo/keto reductase; *f*, NAD^+^-binding d-isomer specific 2-hydroxyacid dehydrogenase

Fructose metabolism in *C. salexigens* has not been described previously. Two pathways for fructose incorporation into central metabolism are predicted based on genome annotation [26]. The first pathway, resembling the Embden–Meyerhof–Parnas pathway (EMP-like), involves the entrance of fructose through a sugar specific phosphotransferase system (PTS^Fru^) with fructose-1-phosphate as the first committed metabolic intermediate. The second pathway involves an unidentified non-PTS sugar transporter (either an ABC transporter for sugars or a facilitator, Fig. 3a), and the activation of fructose to fructose-6-phosphate by a soluble kinase, resembling the initial steps of the Entner–Doudoroff pathway (ED-like) for glucose catabolism.

To gain understanding of these pathways, we analyzed the *C. salexigens* genes which products are annotated as enzymes for fructose metabolism, as well as enzymes linking fructose metabolism with central pathways (Fig. 3a, b).

#### The Embden–Meyerhof–Parnas-like pathway

First, the EMP-like pathway was investigated. The three-gene cluster *csal2646*–*csal2648* has high similarity to the *fruBKA* operon of various *Pseudomonas* species. The *fruB*- and *fruA*-homologous genes *csal2646* and *csal2648* may encode a PTS system specific for fructose import, which phosphorylates this sugar to fructose-1-phosphate [27]. It is noteworthy, fructose is the only sugar for which a specific PTS permease is predicted in *C. salexigens* genome. The *csal2647* gene, homologous to *fruK* from *P. putida*, may encode a soluble 1-phosphofructokinase, which phosphorylates fructose-1-phosphate to form fructose-1,6-bisphosphate [11]. The fructose-1,6-bisphosphate aldolase Ald, (*csal0370*), which splits fructose 1,6-bisphosphate into the common intermediaries dihydroxyacetone phosphate and glyceraldehyde 3-phosphate, connects fructose to central metabolic pathways (Fig. 3a).

#### The Entner–Doudoroff pathway

As stated in the introduction, in order to be metabolized through the ED pathway, fructose has to be activated through a route not involving a PTS system [25]. Fructokinase catalyzes fructose activation to fructose-6-phosphate. Interestingly, the fructokinase gene *frk* (*csal0931*) was found at the 3′-end of a five-gene cluster also encoding the phosphoglucose isomerase (Pgi, *csal0932*, which connects the metabolisms of fructose and glucose), as well as the ED pathway enzymes glucokinase Glk (*csal0935*) and 6-phosphogluconate dehydratase Edd (*csal0936*). The genes involved in the ED route from fructose to glyceraldehyde-3-phosphate are shown in Fig. 3.

#### Analysis of the expression of selected genes of fructose metabolism

To support this in silico reconstruction, we selected a number of key genes for expression analysis. Among the selection, several genes from both fructose metabolic pathways, and a few key genes representative of the connection of fructose and glucoses metabolism, were included. Gene expression levels were assessed on fructose or glucose as sole carbon sources and were normalized to their respective expression levels in glycerol-grown cultures.

**Fig. 4:**
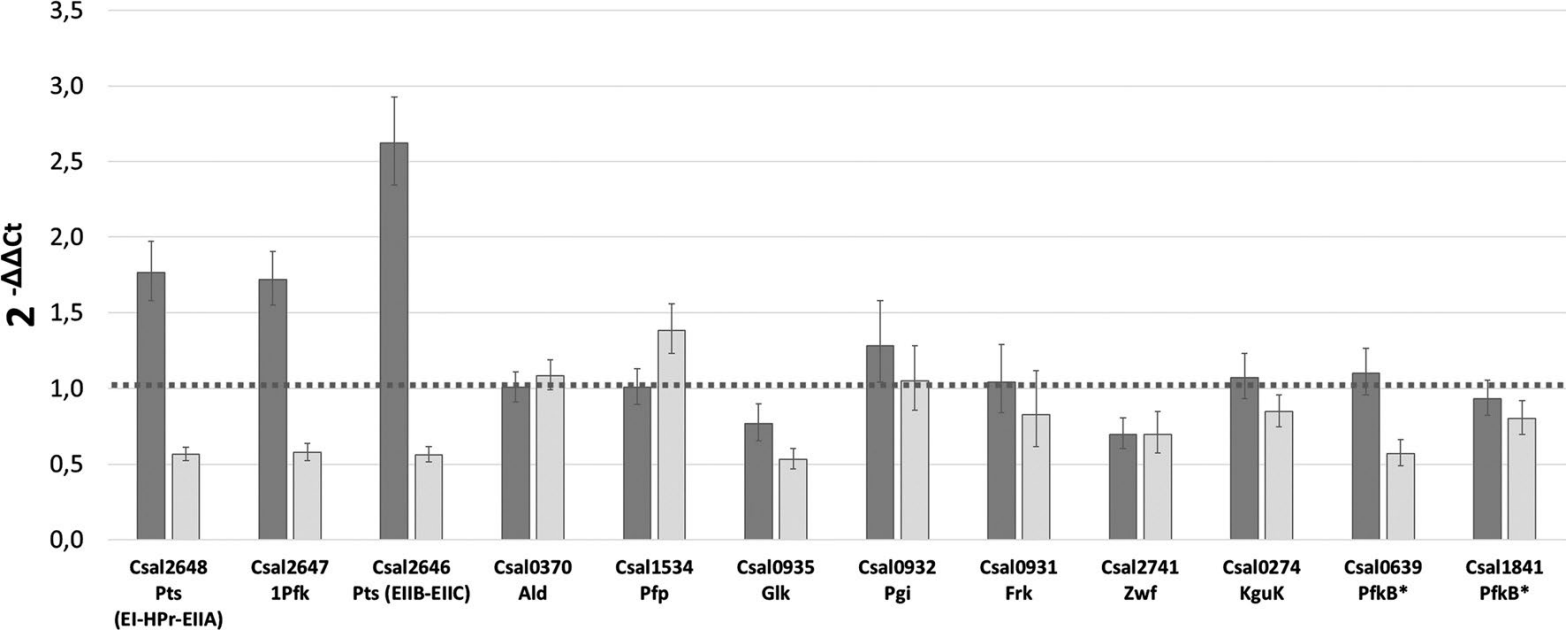
Differential expression of selected genes in fructose- (dark grey) or glucose- (light grey) grown cultures. Relative gene expression was analyzed by RT-PCR of RNA samples were taken from cultures at the exponential phase of growth. ΔΔC_T_ values were calculated taking glycerol-grown cultures as reference condition and the results are expressed as $2^{{ - \Delta \Delta {\text{C}}_{\text{T}} }}$, calculated as the averages of at least two biological replicates and three technical replicates. The *polA* and *dnaA* genes from *C. salexigens*, encoding DNA polymerase I and DNA-binding transcriptional dual regulator, respectively, were used as internal control for relative quantification. See “Methods” for further details. The abbreviations for the names of the enzymes are given in Fig. 3 legend. *Csal2648* sequence comprises domains orthologue to subunits EI, HPr, and EIIA of the fructose specific phosphotransferase system (Pts^Fru^). *Csal2646* sequence comprises domains orthologue to subunits EIIB and, EIIC of the fructose specific phosphotransferase system (Pts^Fru^). *Automatic assignments still needing experimental evidence

As shown in Fig. 4, growth in fructose minimal medium resulted in increased expression of the three genes of the *fruBKA* operon (*csal2646*–*csal2648*). Conversely, these genes were down-regulated by 40% in glucose cultures compared to the expression levels observed in cells grown in glycerol, further reinforcing their specific role in the EMP-like pathway for fructose metabolism in *C. salexigens* (Fig. 3a).

The expression of all other genes analyzed was unaffected, indicating the carbon sources assayed have no role in the regulation of their expression levels.

#### Functional analysis of Csal2647 (1-Pfk) and Csal0931 (Frk)

The metabolic roles of Csal2647 and Csal0931 were proposed previously based on the analysis of their sequences and genomic environment [11] but experimental confirmation was lacking. To confirm the 1-Pfk and Frk activities of Csal2647 and Csal0931, respectively, their corresponding genes were cloned and expressed in *E. coli* BL21(DE3). The heterologous proteins were affinity-purified and their activities assayed in vitro (see “Methods”).

As expected, Csal2647 showed 1-Pfk activity (Table 1). Some residual Frk activity (two orders of magnitude lower) was also detected, which could be due to the presence of traces of fructose in the fructose-1-phosphate reagent, rather than to wide substrate promiscuity. Only the expected Frk activity was detected for Csal0931 (Table 1).

**Table 1:** Enzyme activities detected by spectrophotometric methods in the three purified proteins expressed in *E. coli* BL21 (DE3) *ΔpatZ* from the corresponding heterologous genes of *C. salexigens*

Enzyme activity	Csal2647	Csal0931	Csal0639
Frk	0.05 ± 0.02	2.71 ± 0.75	ND
1-Pfk	1.12 ± 0.32	ND	ND

Values shown are the average and standard deviation of at least two biological replicates and three technical replicates, and correspond to units of enzyme activity per milligram of protein. Proteins were also assayed for the following enzyme activities (although no detectable activity was observed)

Frk: fructokinase; 1-Pfk: 1-phosphofructokinase; 6-Pfk: 6-phosphofructokinase; Glk: glucokinase; Pfp: pyrophosphate dependent 6-phosphofructokinase; ND: not detected

### Contribution of the ED- and EMP-like pathways to fructose metabolism: insight from ^13^C-NMR and ^31^P-NMR studies

#### Quantification of the relative activity of the ED and EMP-like pathways using ^13^C-NMR

To gain insight into the actual metabolic fate of fructose, tracking of ^13^C from isotopically labeled fructose and glucose was performed. Label at C_1_ of both hexoses was chosen, as this position yields different labeling patterns in ectoines when hexoses are metabolized through the ED- or EMP-like pathways [11]. Accordingly, the methyl group of ectoines was predicted to be labeled only from hexose precursors that are metabolized through the EMP-like pathway (Additional file 1: Table S1).

**Fig. 5:**
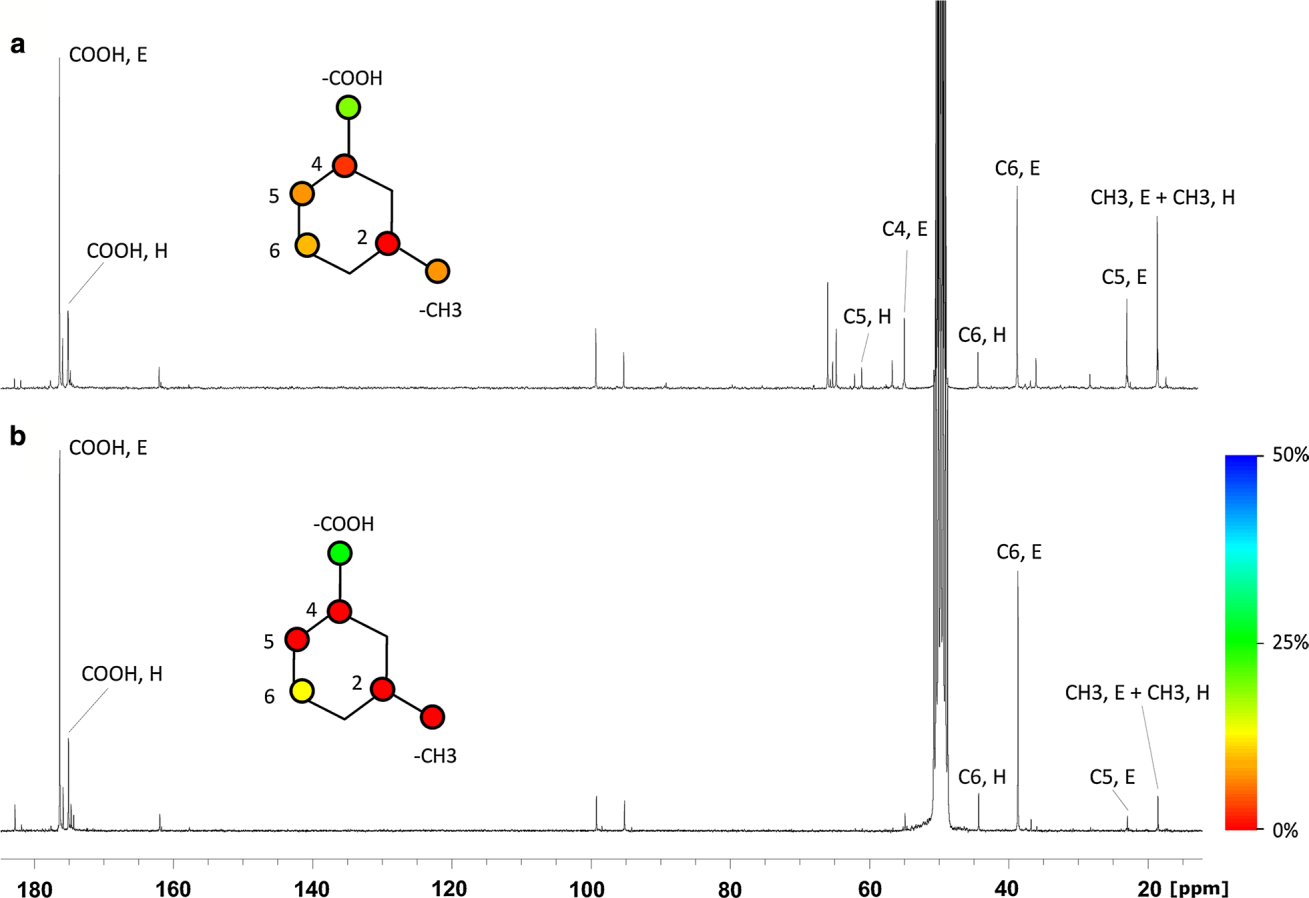
^13^C-NMR spectra of intracellular extracts of **a** [1-^13^C]-fructose and of **b** [1-^13^C]-glucose grown cultures. M63 minimal medium with 0.75 M NaCl and 20 mM of the isotopically labeled carbon source was used. Signals corresponding to labeled carboxylic carbon (COOH), C_4_, C_5_, C_6_ and methyl group (CH_3_) from ectoine (E) and hydroxyectoine (H) are noted. The carbon backbone schemes of ectoines are coloured according to the experimental percentage of ^13^C isotopic abundance at each carbon position of ectoine and hydroxyectoine. Relative labeling for each carbon atom is indicated by the color scale at the right of **b**. Note that largest differences between carbon sources in percentage of labeling are found for C_5_ and CH_3_ positions, the last one being taken as an indicator of EMP flux contribution to the synthesis of ectoines

When growing on fructose as the sole carbon source, the methyl carbon of ectoines exhibited up to 10% of ^13^C label enrichment, both at 0.75 and 2.5 M NaCl. This percentage would involve an 18.8 ± 3.0% contribution of the EMP route to ectoines synthesis from fructose (the rest of fructose being catabolized through the ED pathway). The contribution of other routes, such as the pentose phosphate pathway (PPP), to this labeling was negligible. In contrast, when growing in glucose as the sole carbon source, ectoines showed only 1.2 ± 0.1% of ^13^C enrichment at this position, which is close to the natural ^13^C abundance (Fig. 5 and Table 2, data for cells grown in medium containing 0.75 M NaCl). Thus, the isotopic labeling of ectoines demonstrates that the EMP pathway has a significant contribution for fructose metabolism in *C. salexigens*.

**Table 2:** Percentage of ^13^C-labelling at each of the carbon atoms of ectoines synthesized by *C. salexigens* using either [1-^13^C]-fructose or [1-^13^C]-glucose as the sole carbon source

	Labelled position	[1-^13^C]-fructose	[1-^13^C]-glucose
	CH_3_ (methyl)	8.30 ± 1.76	1.17 ± 0.10
	C_2_	1.65 ± 0.14	1.16 ± 0.28
	COOH	17.14 ± 0.06	28.65 ± 0.85
	C_4_	3.59 ± 0.70	1.16 ± 0.37
	C_5_	7.23 ± 1.76	1.02 ± 0.22
	C_6_	10.04 ± 1.66	14.36 ± 2.11

*C. salexigens* were grown in M63 minimal medium supplemented with 0.75 M NaCl, using either labelled fructose or glucose as the sole carbon source. Fructose cultures were carried out in triplicate; glucose cultures were carried out in duplicate

In order to quantify labelling, percentage of labelling were estimated for each sample analyzed independently from quantitative ^1^H-NMR and ^13^C-NMR spectra. Estimations for ectoine and hydroxyectoine were carried out separately for each condition

#### Detection of key enzyme activities in vitro by ^31^P-NMR

Once that isotopic labeling pattern of ectoines has shown that both the ED and EMP pathways contribute to fructose metabolism, whole cell protein extracts from *C. salexigens* fructose cultures were assayed for various enzyme specific activities. Since all activities tested involved phosphorylated intermediaries and cofactors, ^31^P-NMR was used to monitor their appearance and/or disappearance.

Several different activities were analyzed in cell extracts of *C. salexigens* CHR61 grown in the presence of 0.75 M NaCl. Two of them, fructokinase (Frk, Additional file 1: Figure S2) and phosphoglucose isomerase (Pgi, Additional file 1: Figure S3) (attributable to Csal0931 and Csal0932, respectively) confirmed the direct link between fructose uptake and the ED-like route. 1-Phosphofructokinase (1-Pfk) was also detected (Additional file 1: Figure S4) and accounted for the PTS-dependent, EMP-like route for fructose catabolism. This activity could be attributable to Csal2647 as part of the putative *fruBKA* operon in *C. salexigens*. The detection of the activity of the pyrophosphate dependent 6-phosphofructokinase (PP-Pfk, Additional file 1: Figure S5) will be mentioned in the following section.

### Are ED and EMP pathways connected through phosphofructokinases?

Because fructose is metabolized partially to fructose-6-phosphate (via Frk), conceivably a 6-phosphofructokinase might be involved in the subsequent metabolism of the latter compound. There are two genes in the *C. salexigens* genome, *csal0639* and *csal1841*, that have been annotated to encode 6-phosphofructokinases (PfkB), and one gene, *csal1534*, that has been annotated to encode a putative pyrophosphate-dependent phosphofructokinase (Pfp) (Fig. 3a).

#### Analysis of the roles of putative PfkB-encoding genes csal0639 and csal1841

The expression of *csal0639* and *csal1841* genes was assessed on fructose or glucose as the sole carbon source, using glycerol cultures as reference. While the expression levels of *csal0639* on fructose and glycerol were similar, it was down-regulated in glucose cultures (Fig. 4). The *pfkB*-like gene *csal0639* was cloned, expressed in *E. coli* BL21(DE3) and affinity-purified. The protein was assayed for five enzyme activities: 1-phosphofructokinase, ATP-dependent 6-phosphofructokinase, fructokinase, glucokinase and pyrophosphate dependent 6-phosphofructokinase (glycolysis) and fructose bisphosphatase (gluconeogenesis). Csal0639 exhibited no measurable activity in any of the five assays.

#### Functional complementation of fructose bisphosphatase mutation in *E. coli* by *csal1531*

Pyrophosphate-dependent fructose 6-phosphate-1-kinase activity was detected in *C. salexigens* cell-free extracts using ^31^P-NMR (Additional file 1: Figure S5). The enzyme assay was carried out in the presence of the pyrophosphatase inhibitor NaF and 5 mM pyrophosphate [28, 29]. These conditions might not be physiologically relevant, since the actual pyrophosphate concentration under physiological conditions might not be sufficient to drive the reaction in the kinase direction.

Despite *csal1534* had been initially annotated as pyrophosphate dependent phosphofructokinase (Pfp; EC 2.7.1.90; https://www.ncbi.nlm.nih.gov/protein/ABE58887.1), we have previously proposed that it might actually function in vivo as fructose bisphosphatase on the basis of its sequence similarity to an ortholog from *Methylococcus capsulatus* Bath [11]. The genome of *C. salexigens* does not contain any gene with recognizable similarity to any known Fbp-encoding genes [23]. Moreover, fructose bisphosphatase (Fbp) is required for growth on gluconeogenic carbon sources, and *C. salexigens* can grow on glycerol and a number of other gluconeogenic compounds as sole carbon sources [23].

In order to shed light on this fact, we set out screening *C. salexigens* genome for fructose bisphosphatase encoding genes. We transformed a genomic library of *C. salexigens* DSM 3043 in plasmid pSMART-LCAmp into an *E. coli fbp* mutant and selected derivatives that could grow on glycerol. The DNA sequence of the clone that complemented the *fbp* mutation revealed that it included a string of nucleotides that have a high sequence identity to the *csal1534* gene of *C. salexigens* DSM 3043 (Additional file 1: Figure S6).

**Fig. 6:**
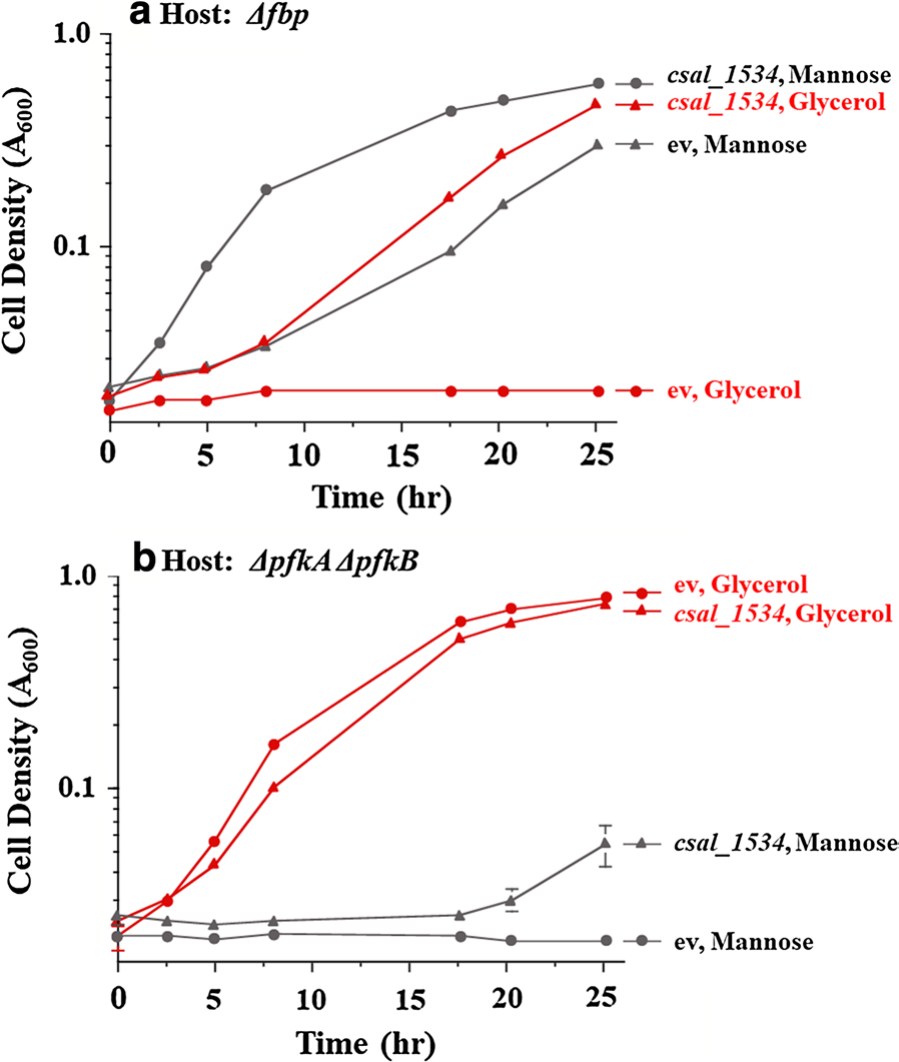
Growth experiments to test functional complementation of fructose bisphosphatase and phosphofructokinase mutations by the *csal15354* gene. Cultures were grown in M9 containing glycerol or mannose as sole carbon sources as indicated, plus kanamycin. **a** Host strain was *E. coli* JW4191 (Δ*fbp*); **b** host strain was *E. coli* RL257 (Δ*pfkA* Δ*pfkB*). The strains carried the empty cloning vector pSMART-HCKan (ev) or the *csal1534* gene inserted into pSMART-HCKan. The results are the averages of experimental replicates. Where not visible, the experimental variability was less than the size of the symbols

Functional complementation assays were carried out to demonstrate the actual in vivo function of *csal1534*. In *E. coli*, *fbp* mutants are unable to grow on glycerol as sole carbon source [30], and *pfkA pfkB* double mutants, which lack both of the 6-phosphofructokinase isoenzymes of this organism, cannot grow on carbon sources that feed into fructose-6-phosphate, such as mannose [31]. The *csal1534* gene was re-cloned into the high copy plasmid pSMART-HCKan and tested for its ability to complement *fbp* single and *pfkA pfkB* double mutations. As can be seen in Fig. 6, the *csal1534* gene could restore growth on glycerol to the *fbp* mutant but not to the *pfkA pfkB* double mutant. These results demonstrate that the Csal1534 protein can function as fructose bisphosphatase in vivo. A similar finding was demonstrated with the Pfp protein of *Propionibacterium freudenreichii*, which could complement the loss of fructose bisphosphatase in *E. coli* but not the loss of 6-phosphofructokinase [32], demonstrating that Pfp can operate in the gluconeogenic but not in the glycolytic direction in vivo. *P. freudenreichii* Pfp and Csal1534 protein share only very marginal sequence similarity (BlastP similarity Expect value = 5e−13; Additional file 1: Figure S6), suggesting that both proteins probably belong to different families.

The result that the Csal1534 protein cannot function efficiently in the glycolytic direction can account for the fact that glucose is metabolized almost completely by the ED rather than by the EMP pathway in *C. salexigens*. Since fructose can be transformed in *C. salexigens* both into fructose-6-phosphate and fructose-1,6-bisphosphate, it remains an open question whether Csal1534 is required for the metabolism of this sugar via the EMP or via the ED pathways. However, it seems unlikely that after the formation of fructose-1,6-bisphosphate by 1-Pfk, Pfp would carry out a large-scale hydrolysis of this product back to fructose-6-phosphate, because these two opposing reactions would constitute a futile cycle.

## Discussion

Microorganisms have evolved an enormous flexibility in fueling central carbon metabolism [33]. We previously reported the lack of 6-phosphofructokinase activity in *C. salexigens.* As a result of this, glucose is exclusively metabolized through the ED-pathway [11]. In this work, we further confirmed using ^31^P-NMR that *C. salexigens* lacks a glycolytic ATP-dependent 6-phosphofructokinase activity (data not shown) which sums up to previous results. In this work, we have demonstrated that *C. salexigens* has different strategies for the metabolism of glucose and fructose. We identified two routes for the uptake, activation and metabolism of fructose, one of them involving initial activation of fructose through a PTS transporter. As described in the introduction, the transport of sugars through PTS is not compatible with their exclusive metabolism via the ED pathway [25].

When redundancy of metabolic routes exists, pathway usage is finally determined by the environmental pressure faced by the microorganism, since survival and proliferation depend on the optimality of the fluxome distribution and its suitability to satisfy the imposed metabolic needs. The routes for fructose metabolism differ in the overall stoichiometry, with different efficiency in terms of energy and redox balances, which has implications in the environmental adaptability of the microorganism.

*Archaea* possess unique, modified variants of the EMP and ED pathways which differ substantially from those of *Bacteria*. For instance, many *Archaea* lack the PEP phosphotransferase system to transport and activate sugars, and the first step of the EMP pathway is sugar phosphorylation by kinases [34]. Some members of the halophilic archaea *Halobacteriaceae* metabolize fructose almost entirely through the EMP-like pathway, whereas glucose is metabolized preferentially through the ED pathway [35, 36]. Such flux distribution for fructose and glucose metabolism contrasts with that found in *C. salexigens*. However, *Halobacteriaceae* do not accumulate organic compatible solutes [37, 38] and, consequently, their metabolic requirements differ from those of halophilic bacteria.

In the non-halophilic *P. putida* and other *Pseudomonas* fructose is partially metabolized through the EMP pathway [24, 39], most fructose being metabolized through the ED pathway. As in the case of *C. salexigens*, *P. putida* possesses all necessary enzymes to assimilate fructose through the EMP route, including a ^Fru^PTS for fructose transport and activation, and lacks ^Glc^PTS [40]. The reasons to prefer the ED route might be related to their lifestyle, e.g. an increased demand of pathway intermediates for constructing cell wall polymers and/or the increased demand for NADPH [11, 39]. *P. putida* favors the ED pathway over the EMP pathway in order to gear its aerobic metabolism to endure oxidative stress-related insults. Knocking-in 6-phosphofructokinase in *P. putida* did not reconstruct the EMP pathway but actually imbalanced the ATP and NADPH levels, which was detrimental for growth [39].

When growing at high salinity, *C. salexigens* incorporates up to 16% of the hexose supplied into ectoines [11]. The synthesis of ectoines imposes a high metabolic burden due to the high metabolic demands: 2 mol of pyruvate and NADPH are needed per mol of ectoine [11, 12, 41]. In addition, high salinity elicits a strong oxidative stress response in *C. salexigens* [42]. The choice of pathway for sugar catabolism affects the overall stoichiometry of ectoines synthesis. Ectoines synthesis through the EMP pathway is ATP neutral, while through the ED pathway it leads to ATP consumption (see Additional file 1). Sugar oxidation through the ED pathway yields NADPH which, accordingly, means a competitive advantage at high salinity. Sugar oxidation through the EMP pathway has higher ATP yield, which compensates its net consumption in the ATP-demanding ectoines biosynthesis. By balancing the fluxes through EMP and ED pathways, *C. salexigens* might offset energy and NADPH demands, highlighting the advantage of redundancy in the pathways.

Other hypotheses explaining why the ED pathway could be preferred have been recently proposed. The ED route is more efficient than EMP in terms of enzymatic protein mass needed to metabolize glucose [18]. Such study proposes this as an explanation why a high proportion of aerobic organisms use exclusively the ED route for glucose oxidation compared to facultative anaerobes; once a non-glycolytic ATP source is assured (oxidative phosphorylation), the ED route may be considered preferable under certain circumstances. Moreover, the synthesis and accumulation of ectoines in *C. salexigens* is partly supported at the expense of total cellular protein [11], which could also contribute to the choice for the ED route.

Very recently, a cycle involving the joint operation of the ED and EMP pathways has been described in glucose-grown *P. putida* [43], which results in increased NADPH synthesis at the expense of net ATP formation. Thus, regardless of the carbon source used, the metabolic network of *P. putida* seems to be constrained by NADPH requirements that must be met. This reflects metabolic adaptations that microbes requiring high amounts of reducing equivalents, such as the case of *P. putida* and *C. salexigens* [11, 39] could have evolved, compelled by their lifestyles.

Summarizing, there are two alternative and interconnected pathways for fructose incorporation in *C. salexigens*, the ED- and EMP-like routes, which differ in their energy and redox yields. Enzyme activities of these pathways have been demonstrated by ^31^P-NMR and, in the case of Csal0931 (Frk) and Csal2647 (1Pfk), by biochemical assays; the role of Csal0639 remains to be uncovered. The protein encoded by *csal1534* gene, originally annotated as a pyrophosphate-dependent phosphofructokinase, is a gluconeogenic fructose bisphosphatase in vivo. This is the only enzyme with this activity found in *C. salexigens* genome and it would be the eighth different mode of known fructose bisphosphatase activity. Finally, the ratio of carbon fluxes split between both routes was estimated by tracing isotopic label by means of ^13^C-NMR and the preference for the usage of the ED pathway was underlined.

## Conclusions

Overall, the existence of two alternative pathways for fructose metabolism in *C. salexigens* has been demonstrated. This conferred this bacterium a way of reconciling the high biosynthetic demands imposed by environmental constraints with its own needs for growth and maintenance. The specific mechanisms that link the metabolic flux ratio through ED and EMP-like routes with overflow metabolism suppression and balance of anaplerotic and TCA fluxes remain to be uncovered.

## Methods

### Bacterial strains and cultures

*Chromohalobacter salexigens* CHR61, a spontaneous rifampicin-resistant mutant of *C. salexigens* DSM 3043^T^ (Table 3), was used throughout this study. Glycerol stocks, solid culture media, and precultures were supplemented with rifampicin to a final concentration of 25 µg/mL. Precultures were started from frozen 20% glycerol stocks and grown in SW-2 medium (containing 2% (w/v), or 0.3 M, total salts) composed of: 5 g/L of yeast extract, 15.6 g/L NaCl, 4.07 g/L MgSO_4_·7H_2_O, 2.6 g/L MgCl_2_·6H_2_O, 0.4 g/L KCl, 67 mg/L CaCl_2_·2H_2_O, 47 mg/L NaBr, and 13 mg/L NaHCO_3_ [44].

**Table 3:** Strains and plasmids used in this work

Strains	Relevant genotype/use	Reference or source
*Chromohalobacter salexigens* CHR61	Spontaneous Rf^r^ mutant of DSM 3043	Cánovas et al. [54]
*E. coli* Top10F’	Host for pRSETB and pRSETC plasmids propagation	Invitrogen
*E. coli* BL21(DE3) Δ*patZ*	Host for heterologous protein expression; F^−^ *ompT hsdS*_*B*_(*r*_*B*_- *m*_*B*_-) *gal dcm λ(DE3) tonA* Δ*patZ*	Castaño-Cerezo et al. [53]
*E. coli* JW4191-1	*fbp*-779::*kan*	Baba et al. [51]
*E. coli* RL257	Δ*pfkA* Δ*pfkB*	Lovingshimer et al. [52]
Plasmids	Cloned region	Source
pRSETB		Life Technologies
pRSETBCsal0639p		This work
pRSETC		Life Technologies
pRSETCCsal0931p		This work
pRSETCCsal2647p		This work

For ectoine production and for the characterization of metabolic pathways, *C. salexigens* was grown in M63 minimal medium (pH 7.2) containing: 13.6 g/L KH_2_PO_4_, 4.2 g/L KOH, 2 g/L (NH_4_)_2_SO_4_, 39.5 mg/L MgSO_4_·7H_2_O, 0.5 mg/L FeSO_4_·7H_2_O. M63 was supplemented with 43.8, 87.6 or 146.0 g/L NaCl (corresponding to 0.75, 1.5 or 2.5 M). As carbon source, 20 mM fructose or glucose was used. M63 cultures were inoculated to an initial absorbance (*A*_600_) of 0.025 with an exponential phase pre-culture grown overnight in SW-2 medium. Batch 100 mL cultures were grown in 0.5 L flasks at 37 °C with aeration on a rotary shaker at 210 rpm.

### Analytical procedures

#### Cell growth

To measure cell concentration, cells were resuspended in a NaCl solution (0.75 to 2.5 M), and absorbance was measured at 600 nm (Novaspec Plus Visible Spectrophotometer, Amersham Biosciences, Little Chalfont, UK). *A*_600_ and dry cell weight (g_DCW_/L) were correlated for the strain used. A separate correlation was established for each salinity used throughout this study: g_DCW_/L = 0.597·*A*_600_; 0.560·*A*_600_ and 0.532·*A*_600_ for 0.6, 0.75 and 2.5 M of NaCl, respectively.

#### Determination of extracellular fructose and organic acids

Extracellular organic acids (gluconate, pyruvate and acetate) and fructose were determined by ion exchange chromatography in a Shimadzu LC-10 HPLC (Shimadzu Scientific Instruments, Columbia, MD) equipped with differential refractive index and diode array (UV) detectors (Shimadzu Scientific Instruments, Columbia, MD). A cation exchange ICE-COREGEL 87H3 column (Transgenomic, Omaha NE, USA) was used for the separation of organic acids. The mobile phase was 15 mM H_2_SO_4_ at a 0.5 mL/min flow rate and 45 °C.

#### Determination of glucose consumption

Glucose was assayed by a glucose (hexokinase) assay kit (Sigma, Saint Louis, MO, USA), using a 96-well microplate reader Synergy HT (Bio-Tek, Winooski VT, USA).

### RNA extraction and RT-PCR

Relative gene expression was analyzed from *C. salexigens* cells cultured in M63 minimal medium with 0.75 M NaCl with 20 mM glucose, fructose or glycerol as the sole carbon source. Cultures were sampled in the late exponential growth phase.

Total RNA was isolated from 3 × 10^8^ cells. Qiagen RNeasy^®^ Mini Kit was used according to the manufacturer’s instructions. The RNA extracts were treated with RNase-free DNase (Qiagen, Venlo, Netherlands) to avoid DNA interferences during polymerase chain reaction (PCR). Purity and concentration were assessed with a NanoDrop^®^ ND-1000 spectrophotometer (NanoDrop Technologies, Wilmington, DE, USA), and quality was evaluated on an Agilent 2100 Bioanalyzer (Agilent Technologies, Palo Alto, CA, USA) using the Agilent RNA 6000 Pico kit. Isolated RNA was stored at − 80 °C for no more than 3 days. One microgram of high-quality RNA (rRNA ratio [23S/16S] ≈ 1.6, RNA integrity number [RIN] > 9.0, and A_260_/A_280_ ratio > 2.0) was reverse-transcribed with TaqMan^®^ Reverse Transcription Reagents (Applied Biosystems, Foster City, CA, USA) and stored at − 20 °C until used. A 50 μL reaction mixture was incubated in a PCR-Thermal Cycler 200 (MJ Research Inc., Boston, MA, USA) for 10 min at 25 °C, 30 min at 48 °C, and 5 min at 95 °C. The primers used in this work (listed in Additional file 1: Table S2) were designed using the Primer Express^®^ Software v3.0 (Applied Biosystems) and ordered from Sigma (Saint Louis, MO, USA). The *polA* and *dnaA* genes from *C. salexigens*, encoding DNA polymerase I and DNA-binding transcriptional dual regulator, respectively, were used as internal control for relative quantification. Quantitative PCR was performed on a 7300 real-time PCR System (Applied Biosystems), using Power SYBR^®^ Green PCR Master Mix (Applied Biosystems, Cheshire, UK). 50 μL reaction mixtures containing 10 ng template cDNA and 15 pmol of each primer, were incubated for 2 min at 50 °C, 10 min at 95 °C, and 40 cycles (15 s at 95 °C and 1 min at 60 °C). The linear dynamic range and amplification efficiency of each gene were checked according to manufacturer’s protocol. Raw data were transformed into threshold cycle (C_T_) values using 7300 System SDS v1.3.1 software (Applied Biosystems). Relative gene expression for each culture condition, compared with the reference culture condition, was calculated by the comparative C_T_ method, and results are reported as $2^{{ - \Delta \Delta {\text{C}}_{\text{T}} }}$.

### Isotopic labeling studies and NMR spectroscopy

For the labeling experiments, cells were grown in 100 mL of M63 medium with 0.75 or 2.5 M NaCl in the presence of 100% isotopically labeled [1-^13^C]-glucose or [1-^13^C]-fructose (CortecNet, Voisins-Le Bretonneux, France). Cultures were harvested in mid to late exponential phase (A_600_ 1.5 to 2), and separated from supernatants by centrifugation (16,000×*g*, 15 min, 4 °C). Compatible solutes were extracted from cell pellets by a variation of the method of Bligh and Dyer [45]. Cells were collected by centrifugation and stored at − 20 °C until extracted. Cell pellets were dried in a Heto Vac VR-1 speedvac (Heto Lab Equipment, Allerod, Denmark), resuspended in 250 µL of methanol/chloroform/water mixture (10/5/4, v/v/v) and incubated for 15 min at 37 °C. To extract ectoines and precipitate proteins, 65 µL of chloroform and 65 µL of water were added. Liquid phases were separated by centrifugation and aqueous phase was separated, vacuum dried and reconstituted in deuterated methanol.

^1^H-NMR and ^13^C-NMR spectra were recorded at 25 °C using Brucker AV400 and Brucker AV500 spectrometers at 400 and 500 MHz, respectively, and a relaxation time of 1 s in the case of ^1^H-NMR and 60 s for ^13^C-NMR. Peak areas were integrated for quantification. For ^1^H-NMR peak quantification formic acid was added as external standard to every sample to a final concentration of 5 mM. For ^13^C-NMR peak quantification, a capillary containing [U-^13^C_4_]-1,1′,3,3′-tetramethylurea was introduced in the NMR tube as external standard so that the concentration of ^13^C in the NMR tube was equivalent to 18.1 mM.

### Prediction of the fates of isotopic labels

[1-^13^C]-fructose and glucose were selected for interrogating the relative importance of different pathways of central metabolism, as described previously [46, 47]. The patterns of incorporation of the isotopic label from sugars into ectoines via EMP-like or the ED-like pathways were predicted as previously described [11] (Additional file 1: Figure S1).

### Spectrophotometric enzyme assays

Enzyme assays were optimized for the conditions, media, and strain used in this work. Measurements were carried out in a 96-well microplate reader Synergy HT (Bio-Tek, Winooski, VT). A unit of enzyme activity was defined as micromoles of substrate consumed or product formed per min and was normalized to milligrams of protein (units/mg). In each case, culture samples were withdrawn, and cells were centrifuged (16,000×*g*, 15 min, 4 °C) and resuspended in 65 mM phosphate buffer (pH 7.5). Cells were sonicated on ice with a 3-mm diameter probe using a Vibra Cell VC 375 ultrasonic processor (Sonics Materials, Danbury, CT) and centrifuged (16,000×*g*, 20 min, 4 °C). The supernatant (cell-free extract) was used for subsequent activity measurements. Protein concentration in cell-free extracts was determined by the bicinchoninic acid (BCA) method (BCA Protein Assay kit, Pierce). Enzyme activities were determined spectrophotometrically in a Synergy™ HT Multi-Mode Microplate Reader (BIO-TEK, Winooski, VT) with 200 μL total reaction volume, 40 μL of which corresponded to purified protein solution conveniently diluted with the suitable reaction buffer.

#### 6-Phosphofructokinase (6Pfk)

The method was that of Peng and Shimizu [48] with slight modifications. The measurement buffer was 50 mM imidazole–HCl (pH 7), and the reaction mixture contained 5 mM MgCl_2_, 1 mM NADH, 1 mM EDTA, 5 mM ATP, 2 U/mL aldolase, 8 U/mL triose-phosphate isomerase, 2 U/mL α-glycerolphosphate dehydrogenase and 5 mM fructose 6-phosphate. The enzyme activity was followed for 5 min as the decrease in NADH absorbance at 340 nm (ε_NADH_ = 6220 M^−1^ cm^−1^).

#### 1-Phosphofructokinase (1Pfk)

A modification of the method for 6Pfk activity was used. The measurement buffer was 50 mM imidazole–HCl (pH 7), and the reaction mixture contained 5 mM MgCl_2_, 1 mM NADH, 1 mM EDTA, 5 mM ATP, 2 U/mL aldolase, 8 U/mL triose-phosphate isomerase, 2 U/mL α-glycerolphosphate dehydrogenase and 5 mM fructose 1-phosphate. The enzyme activity was followed for 5 min as the decrease in NADH absorbance at 340 nm (ε_NADH_ = 6220 M^−1^ cm^−1^).

#### Pyrophosphate dependent 6-phosphofructokinase, (PP-6Pfk)

A slightly modified method was applied [49]. The measurement buffer was 50 mM imidazole–HCl (pH 7), and the reaction mixture contained 5 mM MgCl_2_, 0.2 mM NADH, 1 mM EDTA, 2 U/mL aldolase, 3 U/mL triose-phosphate isomerase, 3 U/mL α-glycerolphosphate dehydrogenase and 2 mM fructose 6-phosphate and 1 mM MgPP_i_. The enzyme activity was followed for 5 min as the decrease in NADH absorbance at 340 nm (ε_NADH_ = 6220 M^−1^ cm^−1^).

#### Fructose bisphosphatase (Fbp)

A variation of a previous method was used [50]. The reaction mixture consisted in 100 mM Tris–HCl (pH 7.5), 10 mM MgCl_2_, 50 mM KCl, 2 mM NADP^+^, 4 U/mL glucose 6-phosphate dehydrogenase, 4 U/mL phosphoglucose isomerase and 5 mM K_2_HPO_4_, and fructose 1,6-bisphosphate as reaction initiator. The enzyme activity was followed for 5 min as the increase in NADPH absorbance at 340 nm (ε_NADH_ = 6220 M^−1^ cm^−1^).

#### Glucokinase (Glk)

The method was that of Peng and Shimizu [48] with slight modifications. The reaction mixture contained 100 mM Tris–HCl buffer (pH 7.5), 10 mM MgCl_2_, 50 mM KCl, 2 mM NADP^+^, 5 mM ATP, 4 U/mL glucose 6-phosphate dehydrogenase and 5 mM glucose. The enzyme activity was followed for 5 min as the increase in NADPH absorbance at 340 nm (ε_NADH_ = 6220 M^−1^ cm^−1^).

#### Fructokinase (Frk)

A modification of the method for Glk activity was used. The reaction mixture contained 100 mM Tris–HCl buffer (pH 7.5), 10 mM MgCl_2_, 50 mM KCl, 2 mM NADP^+^, 5 mM ATP, 4 U/mL glucose 6-phosphate dehydrogenase, 4 U/mL phosphoglucose isomerase and 5 mM fructose. The enzyme activity was followed for 5 min as the increase in NADPH absorbance at 340 nm (ε_NADH_ = 6220 M^−1^ cm^−1^).

### Functional complementation of fructose bisphosphatase mutant in *E. coli*

Genomic DNA was isolated from *C. salexigens* DSM 3043 using a DNeasy kit (QIAGEN) and subjected to a time series of partial digestion using a cocktail of blunt-end generating restriction enzymes. DNA fragments of 2–5 kbp were size-fractionated by gel electrophoresis, purified from the gel material using a QIAQuick kit (QIAGEN), and ligated into the low copy plasmid vector pSMART-LCAmp (Lucigen). The genomic library was transformed into *E. coli* strain JW4191-1 missing the *fbp* gene (*fbp*-779::*kan*) [51] and ampicillin-resistant, kanamycin-resistant colonies were selected on M9 minimal plates containing glycerol as the sole carbon source. Transformants were restreaked on M9 minimal + glycerol plates to verify complementation of the *fbp* deletion. Plasmid DNA was isolated from a single complementing transformant using a QIASpin kit (QIAGEN) and subjected to Sanger-type DNA sequencing.

To confirm that the gene *csal1534* on its own could provide the fructose bisphosphatase function and to test whether it could provide 6-phosphofructokinase activity, the gene along with 141 bp upstream and 95 bp downstream was amplified from *C. salexigens* DSM 3043 genomic DNA by PCR. The amplicon fragment of the proper size was prepared for cloning using the End-It End Repair kit (Lucigen), ligated into the high copy plasmid vector pSMART-HCKan (Lucigen) and transformed into general purpose *E. coli* cells. Plasmid DNA from a transformant colony was isolated, confirmed to contain the *csal1534* gene, and then transformed into two *E. coli* strains: JW4191-1 missing the *fbp* gene (*fbp*-779::*kan*) [51] and RL257 carrying mutations in both the *pfkA* and *pfkB* genes [52], with transformants were selected on M9 minimal plates containing glycerol as the sole carbon source or LB plates containing kanamycin (50 µg/mL), respectively. As a control, vector pSMART-HCKan with no insert was transformed into strain RL257 and transformants were recovered on LB plates containing kanamycin (50 µg/mL). Transformants were tested for their growth phenotypes in liquid M9 minimal medium with either mannose or glycerol as sole carbon sources.

### Cloning, expression and protein purification

The genes *csal2647*, *csal0931* and *csal0639* of *C. salexigens* DSM 3043^T^ were PCR amplified from genomic DNA using the primers shown in Additional file 1: Table S2. The resulting PCR products were digested with *Pst*I/*Hin*dIII (*Kpn*I/*Hin*dIII in the case of *csal0639*) and cloned into the bacterial expression vector pRSETC (pRSETB in the case of *csal0639*) (Life Technologies, Carlsbad, CA, USA), which adds a His_6_-tag to the N terminus of the proteins.

The resulting plasmids were transformed into *E. coli* BL21(DE3) Δ*patZ* [53]. For the expression of His_6_-tagged proteins, the transformed strains were grown overnight at 30 °C in LB to *A*_600_ of 0.5, and 0.5 mM isopropyl-d-thiogalactoside (IPTG) was added, after which the cultures were grown for an additional 3 h. Cells were then harvested by centrifugation and washed three times with 0.9% NaCl and 10 mM MgSO_4_. Cell pellets were resuspended in binding buffer (15.5 mM Na_2_HPO_4_, 4.5 mM NaH_2_PO_4_, 500 mM NaCl and 20 mM imidazole, pH 7.4) and lysed by sonication on ice (three 30 s cycles, power input 3) with a 3 mm diameter probe of a Vibra Cell 375 ultrasonic processor (Sonic Materials, Danbury, CT). Cell debris was removed by centrifugation and protein extract was loaded onto His GraviTrap columns (GE Healthcare, Buckinghamshire, UK). His_6_-tagged proteins were purified according to the manufacturer protocol. Purified proteins containing imidazole were cleaned using PD-10 desalting columns (GE Healthcare, Buckinghamshire, UK), and stored in 65 mM phosphate buffer (pH 7.5) at − 80 °C until used for assays.

## Additional file

**Additional file 1.** Additional Figures S1–S5, Tables S1, S2 and Methods.

## Data Availability

All data generated or analysed during this study are included in this published article [and its additional files].
