# Supplementary material for: Fructose metabolism in Chromohalobacter salexigens: interplay between the Embden–Meyerhof–Parnas and Entner–Doudoroff pathways
Source: Microb Cell Fact. 2019 Aug 13;18:134. doi: 10.1186/s12934-019-1178-x (PMC6692947; doi:10.1186/s12934-019-1178-x)
Supplement: Supplementary file 1 — Additional file 1. Additional Figures S1–S5, Tables S1, S2 and Methods. [file 12934_2019_1178_MOESM1_ESM.docx]

*Submitted to: Microbial Cell Factories*

**Fructose metabolism in *Chromohalobacter salexigens*: interplay between the Embden-Meyerhof-Parnas and Entner-Doudoroff pathways**

José M. Pastor^a^*, Nuno Borges^b^, Juan P. Pagán^a^, Sara Castaño-Cerezo^a^, Laszlo N. Csonka^c^, Bradley W. Goodner^d^, Kathryn A. Reynolds^d^, Luís G. Gonçalves^b^, Montserrat Argandoña^e^, Joaquín J. Nieto^e^, Carmen Vargas^e^, Vicente Bernal^f^*, Manuel Cánovas^a^

**ADDITIONAL FILE**

^a^Dept. de Bioquímica y Biología Molecular B e Inmunología. Facultad de Química. Campus Regional de Excelencia Internacional “Campus Mare Nostrum”. Universidad de Murcia. 30100. Murcia (Spain).

^b^Instituto de Tecnologia Química e Biologica. Universidade Nova de Lisboa. Oeiras (Portugal).

^c^Dept. of Biological Sciences, Purdue University. IN 47907-2064. West Lafayette, (USA).

^d^Dept. of Biology, Hiram College, Hiram, OH 44234 (USA).

^e^Dept. of Microbiology and Parasitology. University of Seville. 41012. Seville (Spain).

^f^Centro de Tecnología de Repsol. Repsol S.A. Calle Agustín de Betancourt, s/n. 28935. Móstoles (Spain).

*Corresponding authors.

E-mail: [josempastor@um.es](mailto:josempastor@um.es)

Phone: +34 868 88 73 97, +34 868 88 52 14

ORCID iD: 0000-0003-4813-1125

E-mail: [vicente.bernal@repsol.com](mailto:vicente.bernal@repsol.com)

ORCID iD: 0000-0003-2482-3477

E-mail: [mcanovas@um.es](mailto:mcanovas@um.es)

Phone: +34 868 88 73 93

ORCID iD: 0000-0003-3254-1736

The present work has been partially funded by the projects: BIO2015-63949-R and BIO2014-54411-C2-1-R (MINECO/ FEDER, EU) and by Fundación Séneca 19236/PI/14 project.

Research in the ITQB was supported by project LISBOA-01-0145-FEDER-007660 (Microbiologia Molecular, Estrutural e Celular) funded by FEDER through COMPETE2020 - POCI and by national funds through FCT- "Fundação para a Ciência e a Tecnologia.

Research in the laboratory of L. N. Csonka was supported by the National Science Foundation grant IOS-1456829.

**Table S1.** Predicted labelling pattern of ectoines for each feasible extreme pathway to ectoines from [1-^13^C]-fructose or [1-^13^C]-glucose. The colour scale depicts the level of labelling at each position for the of ectoines produced by the two alternate metabolic pathways (red 0%, yellow 25%, green 50%, blue 100%). For the shake of clarity, the percentage of predicted labelling at each position is also indicated in the schemes.

| -  COOH  2  4  6  5  -  CH3 | **EMP pathway** | **ED pathway** |
| --- | --- | --- |
| **TCA cycle** |  0%  0%  0%  25%  25%  50% |  0%  0%  0%  0%  0%  0% |
| **Anaplerosis^1^** |  25%  25%  0%  25%  25%  50%  0%  0%  0%  50%  0%  50% |  0%  0%  0%  0%  0%  0%  50%  0%  0%  0%  0%  0% |
| **Anaplerosis^1^ + TCA cycle** |  |  |

*Isotopic label distributions (isotopomers) have been predicted considering only one complete turn of the TCA cycle. In the distributions predicted for [1-^13^C]-fructose and [1-^13^C]-glucose, when further turns of the cycle are taken in account, new isotopomers for ectoines are predicted, including products with more than one label in the carboxylic carbon and C6.

^1^Contribution of Ppc (phosphoenolpyruvate carboxylase) activity to overall anaplerosis was neglected based in previous results (Pastor et al. 2013), and only Pc (pyruvate carboxylase) was taken in account.

**Table S2. List of primers used for RT-PCR and cloning experiments.**

| **Group** | **ORF** | **Gene name^1^** | **Name used in this work** | **E.C. Number^1^** | **Forward and reverse primers** |  |
| --- | --- | --- | --- | --- | --- | --- |
| **Internal control** | Csal0001 | *dnaA* | *dnaA* | NA | 5’-CGGCCAGCAGATGATCCT-3’  5’-CCACCCCGCTGATTTCCT-3’ |  |
|  | Csal0548 | *polA* | *polA* | 2.7.7.7 | 5’-CGACGCCTGGATGGTGAT-3’  5’-CGCCTCCTTGACCTCGAAG-3’ |  |
|  | Csal0274 | *kdgK* | *kguK* | 2.7.1.45 | 5’-ATGAAACGGCCGAAGCTG-3’  5’-GGCGTTTCAGGTCGCCT-3’ |  |
| **ORFs analyzed by RT-PCR** | Csal0370 | *fbaA* | *ald* | 4.1.2.13 | 5’-TGATGCCTTCGACGATTTCTT-3’  5’-AGGAATGGCTGGAAGTCATCAA-3’ |  |
|  | Csal0639 | *pfkB^2^* | *Csal0639* | Unknown | 5’-GAACGACTGGCGGATTGC-3’  5’-GCGGGTGAAGACAGCGAC-3’ |  |
|  | Csal0931 | *scrK* | *frk* | 2.7.1.4 | 5’-GGCTATGCTCAGCGTGGTCT-3’  5’-CATCTGTGCAGCAACAGCCT-3’ |  |
|  | Csal0932 | *pgi* | *pgi* | 5.3.1.9 | 5’-GATGACGCCCTGGACGAAT-3’  5’-CCTGGGCGCACTGATTG-3’ |  |
|  | Csal0935 | *glk* | *glk* | 2.7.1.2 | 5’-CGGACAGCGAAGGATAATGC-3’  5’-GGGCGTTCGACCTTCATG-3’ |  |
|  | Csal1534 | *pfkA* | *pfp* | 2.7.1.90 | 5’-GGTCTTGGGAACGTGAATCG-3’  5’-TCGCAGCTCTCGGAAAAGAT-3’ |  |
|  | Csal1841 | *pfkB^2^* | *Csal1841* | Unknown | 5’-CACGCCGAAAACGCCA-3’  5’-TCGCTGAAGCCGTGATTG-3’ |  |
|  | Csal2646 | *fruA* | *pts *EIIB/C* | 2.7.1.69 | 5’-TCAAGGAGAAGGGCGTTTACA-3’  5’-CGACAATGCGATGCACAAC-3’ |  |
|  | Csal2647 | *fruK* | *1-pfk* | 2.7.1.56 | 5’-CGCCAATTGACCATGCAG-3’  5’-TACCGTGTGTGCCGGTGA-3’ |  |
|  | Csal2648 | *fruB* | *pts EI/HPr/IIA* | 2.7.1.69 | 5’-TCGACCTATCTCGGCAATGG-3’  5’-AACTGCAACACCCGTACGC-3’ |  |
|  | Csal2741 | *zwf* | *zwf* | 1.1.1.49 | 5’-TCACGGCGGACGAACAA-3’  5’-GATGCCTACGAGCGTCTGTTG-3’ |  |
| **Primers used for cloning** | Csal0639 | | 5’- GGTGGTGGTACCATGAGCGCAGACCTGCCTC-3’  5’-GGTGGTAAGCTTTCATCGGGCACCTCCGTGG-3’ | | |  |
|  | Csal0931 | | 5’-GGTGGTCTGCAGATGACCCCGCTCATTGCCTTCGG-3’  5’-GGTGGTAAGCTTTCAGGTCATGGCGTCGAGATCGTC-3’ | | |  |
|  | Csal2647 | | 5’-GGTGGTCTGCAGATGGCGCGCGTGCTGACATTGAC-3’  5’-GGTGGTAAGCTTTCATAGGGTCTCCCTCGCGCCG-3’ | | | |

*^1^According to the function associated to the KO Orthology Number assigned in KEGG*

*^2^Provisional assignment given by automatic annotation according to JGI and KEGG*

*NA: Not Applicable*

**Figure S1. Scheme of the Embdem Meyerhof Parnas and Entner Doudoroff pathways in bacteria.** In the EMP pathway, glucose is transported and activated (phosphorylated) by the glucose-specific PEP phosphotransferase system (^Glc^PTS). This system consumes one mol of PEP per mol of glucose transported (activated). In the ED pathway, glucose is activated by an ATP-dependent kinase. The two enzymes which are exclusive of the ED pathway (Edd and Eda) are highlighted in red. The rest of enzymes (shown in blue) are shared with the pentose phosphate pathway (oxidative) or the EMP pathway. The steps where there is consumption or production of ATP or redox cofactors (NADH or NADPH) are indicated. The overall yield of the pathways are shown at the bottom of the scheme.

This scheme depicts the EMP pathway in *Escherichia coli*. As described in previous works, *C. salexigens* lacks ^Glc^PTS and 6-phosphofructokinase and, consequently, all glucose is metabolized through the ED pathway (see Pastor et al. 2013).


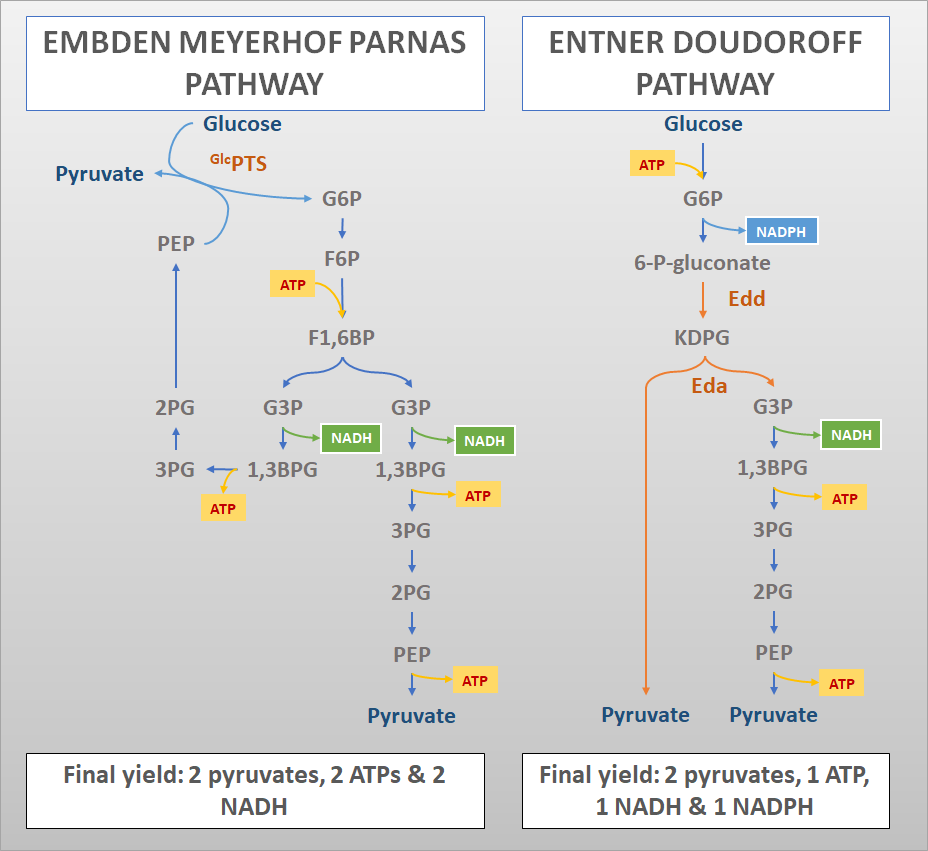


**Global stoichiometric equations of ectoine production from fructose or glucose.**

In order to further understand the differences in the production of ectoines from glucose and fructose, global (lumped) equations were derived for the synthesis of these compounds from the two sugars considered in this work. The overall stoichiometry of the biosynthesis was highly dependent on the pathways followed for sugar metabolism.

1. ***Production of ectoine from glucose through the ED pathway (with periplasmic initial oxidation of glucose):***

Glucose + 2·NH_4_^+^ + ATP + 2·NAD^+^ + 3·NADPH + quinone 🡪 Ectoine + ADP + 2·NADH + 3·NADP^+^ + hydroquinone + Pi + 3·H_2_O + H^+^

1. ***Production of ectoine from glucose or fructose through the ED pathway (with cytosolic initial oxidation of glucose or fructose):***

Glucose/Fructose + 2·NH_4_^+^ + ATP + 2·NAD^+^ + 2·NADPH 🡪 Ectoine + ADP + 2·NADH + 2·NADP^+^ + Pi + 3·H_2_O + 2·H^+^

1. ***Production of ectoine from fructose through the EMP pathway:***

Fructose + 2·NH_4_^+^ + 3·NAD^+^ + 3·NADPH 🡪 Ectoine + 3·NADH + 3·NADP^+^ + 4·H_2_O + 2·H^+^

From these overall equations, the following conclusions can be drawn:

- The production of ectoine from glucose/fructose involving the ED pathway is ATP consuming.
- The production of ectoine from fructose through the EMP pathway is ATP-neutral.
- The production of ectoine from fructose involving the EMP pathway involves a higher consumption of NADPH equivalents than through the ED pathway.

Therefore, balancing the flux ration between the ED and EMP pathways for the production of ectoines from fructose allows *C. salexigens* to enhance ATP production at the expense of consuming a higher amount of NADPH. This could be contributing to a higher ectoine yield and more efficient metabolism (with a lower rate of carbon overflow) during growth in fructose cultures.

**ADDITIONAL METHODS**

***Enzyme Assays: enzyme activity detection by ^31^P-NMR***

Cell extracts were obtained from 800 mL cultures of *C. salexigens* grown in M63 medium supplemented with NaCl 0.75 M and fructose 20 mM as sole carbon source. Samples were collected for centrifugation at late exponential phase (A^600^ ~ 2). Cells were sonicated on ice with a 3-mm diameter probe using a Vibra Cell VC 375 ultrasonic processor (Sonics Materials, Danbury, CT, USA) and centrifuged (16,000x*g*, 20 min, 4 °C). Cell extracts were desalted and small organic compounds removed using PD-10 gel permeation desalting columns (GE Healthcare, Buckinghamshire, UK), to avoid enzyme activity inhibition and/or interferences. These cell-free extracts were used for subsequent activity measurements.

Reactions were initiated by the addition of protein extract to a mixture of a final volume of 500 µL. Reactions were immediately monitored by ^31^P-NMR. For every experiment, spectra were recorded as cumulative signal of periods of 20-30 minutes. Temperature was kept at 30°C in every case. When ATP or pyrophosphate was added as substrate, NaF was also added to inhibit ATPases. After running each spectra series, extracts were heated 10 min at 85°C for complete enzyme inactivation. EDTA was added to a final concentration of 50 mM to chelate magnesium ions and improve spectra resolution. After addition of EDTA, extracts were spiked with the corresponding pure compounds to confirm their identity. Reaction mixtures contained for the following enzymes:

1. *1-Phosphofruktokinase (1Pfk).* Tris-HCl buffer 100 mM (pH 7), fructose-1-phosphate 5 mM, ATP 5 mM, NaF 30 mM, MgCl_2_ 50 mM and KCl 250 mM.
2. *Phosphoglucose isomerase (Pgi)*. MOPS buffer 100 mM (pH 6.5), fructose-6-phosphate 5 mM, MgCl_2_ 10 mM and KCl 50 mM.
3. *Fructokinase (Frk)*. Tris-HCl buffer 100 mM (pH 7.5), fructose 5 mM, ATP 5 mM, NaF 30 mM, MgCl_2_ 50 mM and KCl 250 mM.
4. *Pyrophosphate dependent 6-phosphofructokinase (PP-6Pfk)*. MOPS buffer 100 mM (pH 6.5), fructose-6-phosphate 5 mM, pyrophosphate 5 mM, NaF 30 mM, MgCl_2_ 10 mM and KCl 50 mM.

**Figure S2.** **Fructokinase activity.** The enzyme was assayed in an extract of *C. salexigens* CHR61 grown in M63 minimal medium with 0.75 M NaCl and fructose 20 mM as the sole carbon source. Protein extracts were obtained from cells harvested during exponential phase of growth and separated from non-proteinaceous impurities. Fructose and ATP were added as reaction substrates to protein extract in a suitable buffer (see Materials and Methods section for further details). Spectra are arranged in time series, from bottom to top: immediately after substrates addition, and after 30, 60 and 90 minutes of incubation. Signals are identified with lower case letters as follows: **a**, fructose-6-phosphate; **b**, ATP and **c**, ADP.

Fructose-6-phosphate-specific NMR signals increased during the assay, thus demonstrating the fructokinase activity assayed.

**Figure S3. Phosphoglucoisomerase activity**. The enzyme was assayed in an extract of *C. salexigens* CHR61 grown in M63 minimal medium with 0.75 M NaCl and fructose 20 mM as the sole carbon source. Protein extracts were obtained from cells harvested during exponential phase of growth and separated from non-proteinaceous impurities. Fructose-6-phoshate was added as reaction substrate to protein extract in a suitable buffer (see Materials and Methods section for further details). Spectra are arranged in time series, from bottom to top: immediately after substrates addition, and after 30, 60 and 90 minutes of incubation. The identity of signals are marked with lower case letters as follows: **a**, glucose-6-phosphate; **b**, fructose-6-phosphate and **c**, phosphate. Glucose-6-phosphate and phosphate specific NMR signals increased while those of fructose-6-phosphate decreased, thus demonstrating the phosphoglucoisomerase activity assayed.


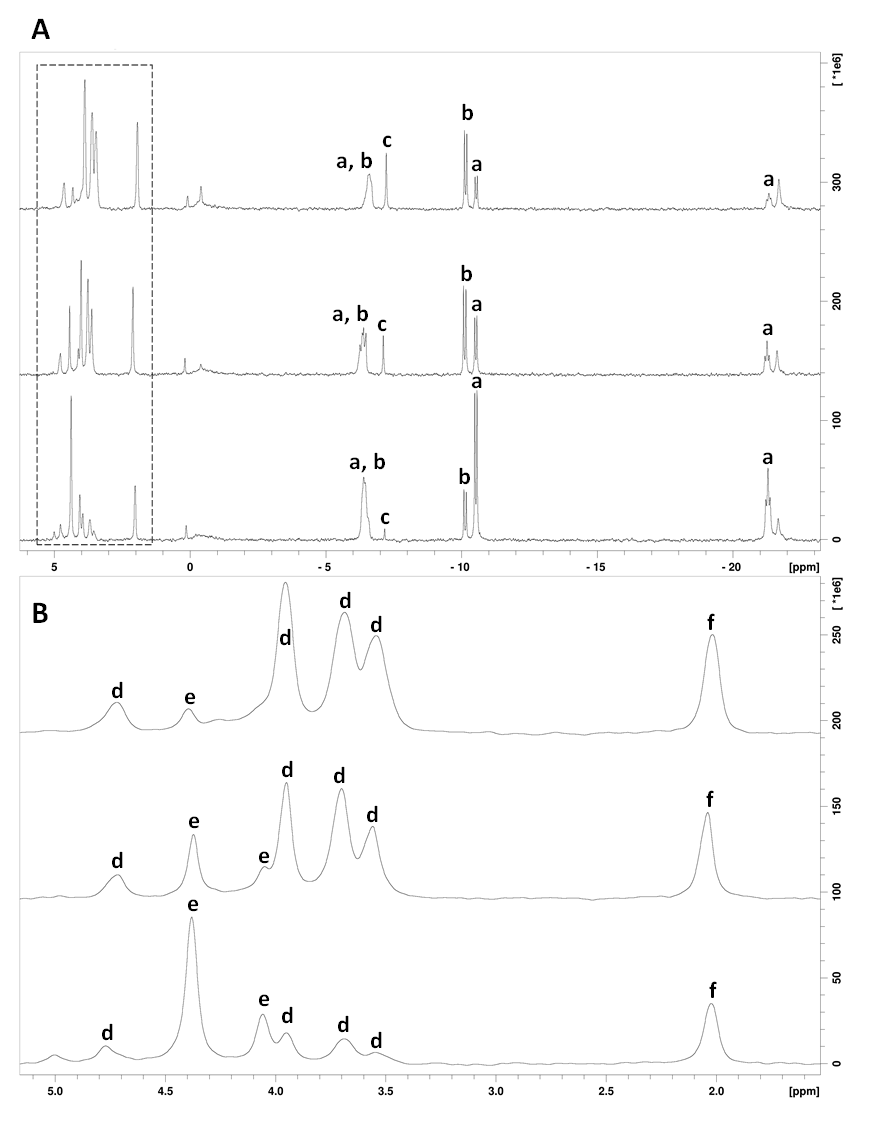


**Figure S4. 1- Phosphofructokinase activity.** The enzyme was assayed in an extract of *C. salexigens* CHR61 grown in M63 minimal medium with 0.75 M NaCl and fructose 20 mM as the sole carbon source. Protein extracts were obtained from cells harvested during exponential phase of growth and separated from non-proteinaceous impurities. Fructose-1-phoshate and ATP were added as reaction substrates to protein extract in a suitable buffer (see Materials and Methods section for further details). **A**, phosphates zone of ^31^P-NMR spectra and **B**, detail of sugar-phosphate zone. Spectra are arranged in time series, from bottom to top: immediately after substrates addition, and after 20 and 40 minutes of incubation. Signals are identified with lower case letters as follows: **a**, ATP; **b**, ADP; **c**, pyrophosphate; **d**, fructose-1,6-bisphosphate; **e**, fructose-1-phosphate and **f**, phosphate.

Fructose-1,6-bisphosphate-specific NMR signals increased while those of fructose-1-phosphate decreased, thus demonstrating the 1-phosphofructokinase activity assayed.


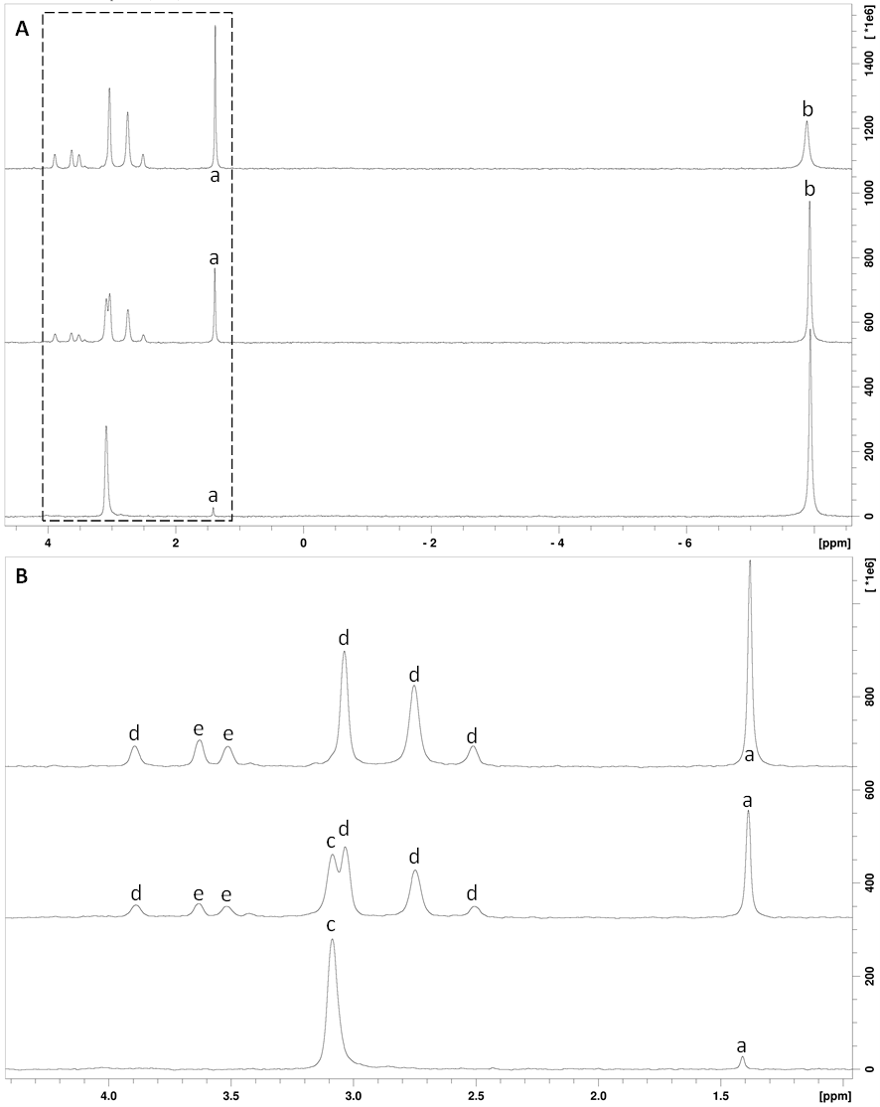


**Figure S5. Pyrophosphate dependent 6-phosphofructokinase activity.** The enzyme was assayed in extracts of *C. salexigens* grown in M63 minimal medium with 0.75 M NaCl and fructose 20 mM as the sole carbon source. Protein extracts were obtained from cells harvested during exponential phase of growth and separated from non-proteinaceous impurities. Fructose-6-phosphate and pyrophosphate were added as reaction substrates to protein extract in a suitable buffer (see Materials and Methods section for further details). A, phosphates zone of 31P-NMR spectra and B, detail of sugar-phosphate zone. Spectra are arranged in time series, from bottom to top: immediately after substrates addition, and after 20 and 40 minutes of incubation.

The identity of signals are marked with lower case letters as follows: **a**, phosphate; **b**, pyrophosphate; **c**, fructose-6-phosphate; **d**, fructose-1,6-bisphosphate; **e**, glucose-6-phosphate.

Phosphate, fructose-1,6-bisphosphate and glucose-6-phosphate-specific NMR signals increased while that of pyrophosphate decreased, thus demonstrating the presence of pyrophosphate dependent 6-phosphofructokinase activity.

**A)**


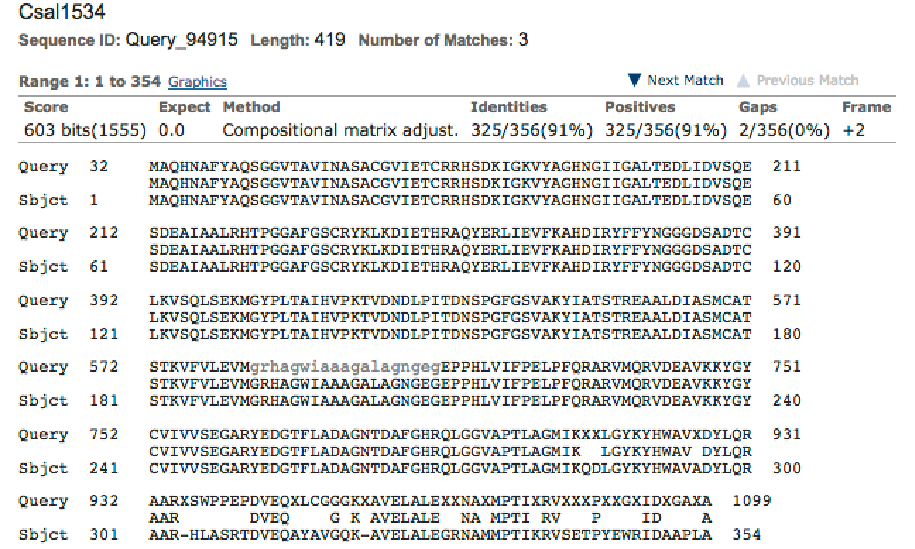


**B)**


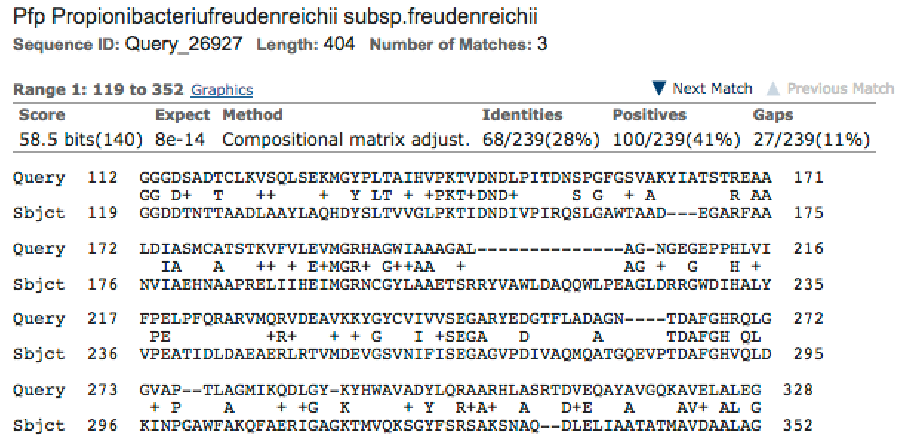


**Figure S6.** **Analysis of the sequence of the *csal1534* gene.** Panel **A)** BlastX comparison of the amino acid sequence of the *fbp*-complementing clone obtained from the genomic library of *C. salexigens* (Query) and the predicted amino acid sequence of the *csal1534* gene (Sbjct). Panel **B)** BlastP comparison of the predicted amino acid sequence of the Csal1534 protein (Query) with that of the *P. freudenreichii subsp. freudenreichii* Pfp protein (Sbjct). Analyses were done using the default settings of the respective Blast algorithms (https://blast.ncbi.nlm.nih.gov/Blast.cgi).
